# A motivational intervention for patients with COPD in primary care: qualitative evaluation of a new practitioner role

**DOI:** 10.1186/1471-2296-15-164

**Published:** 2014-10-06

**Authors:** Susanne Langer, Carolyn A Chew-Graham, Jessica Drinkwater, Cara Afzal, Kim Keane, Cheryl Hunter, Else Guthrie, Peter Salmon

**Affiliations:** Department of Psychology, Manchester Metropolitan University, Manchester, M13 0JA UK; Research Institute, Primary Care and Health Sciences, Keele University, Keele, ST5 5BG UK; Leeds Institute of Health Sciences, University of Leeds, Leeds, LS2 9LJ UK; CHOICE Programme, Manchester Mental Health and Social Care Trust, Manchester, M13 9WL UK; Leeds and York Partnership NHS Foundation Trust, Leeds, LS15 8ZB UK; Health Services Research Unit, Nuffield Department of Population Health, University of Oxford, Oxford, OX3 7LF UK; Manchester Mental Health and Social Care Trust, Manchester, M13 9WL UK; Department of Psychological Sciences, University of Liverpool, Liverpool, L69 3GB UK

**Keywords:** Primary care, Long-term conditions, Qualitative research, Motivational change, Psychosocial intervention

## Abstract

**Background:**

Long-term conditions such as chronic obstructive pulmonary disease (COPD) are growing challenges for health services. Psychosocial co-morbidity is associated with poorer quality of life and greater use of health care in these patients but is often un-diagnosed or inadequately treated in primary care, where most care for these patients is provided. We developed a brief intervention, delivered by ‘liaison health workers’ (LHWs), to address psychosocial needs in the context of an integrated approach to physical and mental health. We report a qualitative study in which we characterize the intervention through the experience of the patients receiving it and examine how it was incorporated into primary care.

**Methods:**

Qualitative study using patient and practice staff informants. We audio-recorded interviews with 29 patients offered the intervention (three had declined it or withdrawn) and 13 practice staff (GPs, nurses and administrators). Analysis used a constant comparative approach.

**Results:**

Most patients were enthusiastic about the LHWs, describing the intervention as mobilizing their motivation for self-management. By contrast with other practitioners, patients experienced the LHWs as addressing their needs holistically, being guided by patient needs rather than professional agendas, forming individual relationships with patients and investing in patients and their capacity to change. Practices accommodated and accepted the LHWs, but positioned them as peripheral to and separate from the priority of physical care.

**Conclusions:**

Despite being a short-term intervention, patients described it as having enduring motivational benefits. The elements of the intervention that patients described map onto the key features of motivating interventions described by Self-Determination Theory. We suggest that the LHWs motivated patients to self-management by: (i) respecting patients’ competence to decide on needs and priorities; (ii) forming relationships with patients as individuals; and (iii) fostering patients’ sense of autonomy. While truly integrated primary care for patients with long-term conditions such as COPD remains elusive, existing practice staff might adopt elements of the LHWs’ approach to enhance motivational change in patients with long-term conditions such as COPD.

**Electronic supplementary material:**

The online version of this article (doi:10.1186/1471-2296-15-164) contains supplementary material, which is available to authorized users.

## Background

In the UK, chronic obstructive pulmonary disease (COPD) is a major challenge for public services in the context of an ageing population, increased demand for services, and continued economic austerity. COPD is the second most common cause of emergency admission and is one of the most costly diseases in terms of acute hospital care – the English NHS spends £810 million on COPD each year, nearly half of which is spent in secondary care [1].

Patients with COPD become inactive, leading to a downward spiral of decline characterised by deconditioning and muscle weakness, reduced exercise capacity and impaired health status [2, 3]. This spiral is likely to be reinforced by the presence of depression or anxiety. One in four patients with COPD will have depression, twice the prevalence in people without COPD. Co-morbid depression and anxiety further reduce quality of life and impair treatment adherence [4] and are associated with even greater use of health services, including unscheduled and primary care [5]. Therefore addressing psychosocial co-morbidity is important, not only in its own right, but also for improving patients’ function and quality of life and, potentially, in reducing or modifying their health care use.

Primary care is regarded as the best setting in which to deliver care for people with COPD and other long-term conditions, reflecting its accessibility and its emphasis on care that is person-centred, integrated and holistic in addressing both physical and emotional health needs [6]. UK government policy has directed and incentivised primary care to improve care for patients with long-term conditions in order to improve their health outcomes and reduce health care costs by transferring health care from hospital services to primary care [7–9]. A key element of this strategy is the Quality and Outcomes Framework (QOF), which financially rewards practices for care in line with clinical quality indicators. However, psychological and social needs in patients with long-term conditions (LTCs) are often missed in primary care patients with LTCs, partly because QOF induces a ‘tick-box’ approach which prioritises biomedical tasks [10]. In addition, patients’ tendency to use normalising attributional styles that see depression as a normal consequence of ill health, along with professionals’ conceptualisations of depression as justifiable and difficult to manage, especially in older adults, are implicated in under-detection of depression in UK primary care [11]. Thus, detection and management of co-morbid depression in people with LTCs by existing primary care practitioners (general practitioners and practice nurses) is poor.

In the USA, collaborative care models that encourage inter-professional working have improved depression outcomes in patients with long-term conditions [12]. However, the attempt to apply these in UK primary care has exposed continuing fragmentation of physical and mental health care [12]. We therefore developed an intervention to promote the management of psychosocial co-morbidity in patients with COPD. Informed by collaborative care [13, 14], whole system frameworks [15, 16] and cognitive-behavioural approaches [12], we introduced a new kind of practitioner in the primary care team to address psychological and social needs and to deliver an integrated approach to physical and mental health care. In this paper, our aims are: to outline the intervention; to use the accounts of patients who experienced the intervention to characterise its main features; to use the accounts of primary care staff to understand how the intervention was incorporated into primary care; and to reflect on implications for meeting psychosocial needs of patients with COPD in UK general practice.

## Methods

A feasibility study as part of a programme of work (CHOICE; NIHR RP-PG-0707-10162). Ethical approval from NRES Committee North West- Greater Manchester East, REC reference: 12/NW/0068.

### Intervention

Two practitioners, whom we called Liaison Health Workers (LHWs), were seconded to the study. Both were female. One had mental health nursing background; the other general nursing and mental health social work. Training, delivered over two days by a professor of liaison psychiatry and professor of mental health, included skills training (patient-centred interviewing and problem solving), psychological interventions, behavioural activation, cognitive restructuring, medication management and liaison skills. Training ended with observed consultations with a simulated patient. In addition, the LHWs shadowed members of the COPD Service at a local hospital, and identified relevant local third-sector services and networks including voluntary groups, Expert Patient Programmes and advice centres. Their work was guided by a treatment manual, which emphasised working with patients to: identify and prioritise psychosocial and clinical needs; address psychosocial needs directly; and liaise with practice staff around clinical needs. They offered self-help booklets and relaxation CDs to patients, sign-posted patients to third sector services, as they judged appropriate, and addressed patients’ social problems (see below). The LHWs received twice-weekly individual or joint supervision from a liaison psychiatrist. Treatment sessions were audio-recorded and re-played in supervision to assess treatment fidelity.

The LHWs were based in the participating general practices, where they had access to electronic patient records on which they documented their activity [12]. They saw patients either in their own homes or occasionally at the practice, or spoke with patients by telephone. They provided up to four sessions, with an option of a further four if required, of up to about one hour each. They chose to wear nurse uniforms and introduced themselves to patients as Liaison Health Workers based in the patient’s general practice.

Participating practices and prospective participant patients received an information leaflet ‘Coping with COPD: How can Liaison Health Workers help?’, individualised with the practice name and LHW contact information, and containing brief information about the LHW and what she could offer. Recruited patients also received a card including a photograph of the LHW and her name and contact information and a plastic wallet to store material provided by the LHWs.

### Participating practices

We used data from Central and South Manchester Primary Care Trusts (now Clinical Commissioning Groups) to target practices with a high prevalence of COPD, recruiting six, of which three were randomised to receive the LHW intervention. Participation in the study was accepted by the Primary Care Trust to meet the requirement for delivery of one of the QP (Quality and Productivity) indicators for the Trust.

The intervention was introduced to practices in one or two (determined by the practices) half-day workshops by a professor of liaison psychiatry and professor of primary care, who outlined the research programme and detailed the intervention. In addition, they discussed with practices the potential to improve internal communications about management of COPD and to improve detection of patients’ psychosocial needs. Each practice was encouraged to consider how to maximise the benefit from the LHWs’ work, and to integrate them as much as possible into practice systems, such as providing a consultation room, filing space, access to practice computer systems (EMIS) to share patient information, and access to team meetings.

### Patient recruitment to the intervention

As a pragmatic trial of a clinical service embedded within GP practices, the inclusion criteria for referral to the LHWs were simple and broad with the specific intention to enhance the external validity of the study. Patients were accepted for treatment if they had a QOF diagnosis of COPD and at least one indicator of psychosocial need, which included a QOF diagnosis of depression, clinical diagnosis of depression by practice staff, social isolation, and chronic or recent psychosocial stressors. Pathways for recruitment included direct referral from GP or other practice staff, and invitation by a letter (enclosing an information leaflet about the LHW service) for patients on the QOF depression register. Of 467 patients on the practices’ COPD registers, 184 were invited to see the LHW (of whom 51 had a QOF diagnosis of depression).

**Figure 1:**
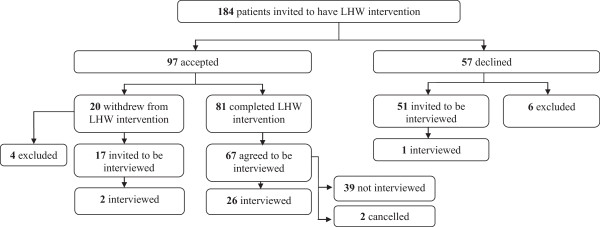
**Participant recruitment.**

Recruitment is detailed in Figure 1. Of 184 patients invited to see the LHW; 97 agreed and 81 completed the intervention (defined as completing 4 meetings or being discharged by mutual agreement after fewer). For recruitment to the qualitative evaluation we adopted maximum variation sampling whereby we sought patients across the range of co-morbidities and ages of those available, at a range of intervals after the intervention ended (from one week to 5 months), and we made strenuous efforts to recruit those who declined or withdrew from the intervention. We excluded patients on a palliative care register. Patients who completed the intervention received a Patient Information Sheet (PIS) describing the qualitative evaluation and a reply slip on which they could indicate willingness to be interviewed and a prepaid envelope. Of 67 patients who indicated willingness to be interviewed, we interviewed 26. Of 57 patients who declined the intervention we sent the PIS and reply slip to 51, of whom one agreed and was interviewed. Of 20 patients who withdrew before completion, we invited 17 to be interviewed, of whom 2 agreed and were interviewed.

We asked staff at the participating practices to participate in interviews. Of 14 general practitioners (GPs), 6 practice nurses (PNs) and health care assistants (HCAs), and 4 administrative staff whom we invited, 5, 4 and 4, respectively, agreed and were interviewed. All participants received written information about the study and provided written consent for interview.

### Data collection and analysis

We used interviews because we wanted to ascertain patients’ and HCPs’ views and experiences. Patients took part in semi-structured interviews (mean 47 minutes) in a private area at the practice or in their homes, as they preferred. Interviews were guided by a topic guide which included: their health difficulties and psychosocial context; expectations of the LHW; experience of the LHW’s involvement, including benefits or difficulties associated with it; comparison with other services currently or previously encountered; and reflections on the intervention over the time since it ended. Practice staff were interviewed in a private area of their practice (mean duration 30 minutes, range 15–40). The interview topic guide included their expectations and experience of the LHWs, including effects on patient care or the practice and (for practitioners) whether and why they referred patients to the LHWs. The researchers conducting the interviews (SL, CH, KK) had previous experience including mental health (all), and long-term conditions and primary care (SL, CH) and were each trained in qualitative interviewing and supervised by the study team. They were independent of the intervention and clinical teams; contact with the LHWs was confined to research tasks. The intervention was time-limited and data collection ended when it came to a close.

Data were anonymised and transcribed verbatim. Analysis was inductive and took a constant comparative approach. The researchers familiarized themselves with the transcripts, and discussion amongst the team identified themes documented in a set of continually updated analysis notes. We paid particular attention to deviant cases that modified our initial analysis. In presenting illustrative data below, we identify participants by numbers. The ellipsis (...) signifies omitted text. Square brackets denote explanatory text. Illustrative quotations are labelled with the practice identifier (R,G,B) and patient identification number and, for staff, the staff category.

To characterise the sample, patients completed two self-report questionnaires at the start of the first meeting with the LHW: PHQ-9 [17] and GAD-7 [18] to assess depression and anxiety, respectively.

## Results

### Sample characteristics

The 29 patients interviewed (16 female) had a mean age of 65 years (range 48–81). In their interviews patients reported a mean of 3.5 conditions in addition to COPD; mainly arthritis, depression, asthma, diabetes and memory problems. Mean PHQ-9 score for interviewed patients was 12.27; mean GAD-7 score was 9.50 (no information is available for those who declined to meet the LHW). These scores were similar to those for the full cohort who accepted the LHW intervention (PHQ-9 mean 11.69; GAD-7 mean 9.08). The 26 patients who completed treatment had a mean of 4.30 treatment sessions (range 3–7).

### Patients’ experiences: a new, motivational intervention

Patients interviewed soon or several months after the LHWs’ involvement provided similar accounts. Almost all were positive about the LHWs, attributing diverse kinds of help to them (Table 1). Comments extended beyond politeness and responses that they might think the interviewer would expect, describing LHWs as, for example, my ‘*angel in the darkness*’_B198_ or having *‘done more than I even dreamt of’*_R195_. Although they knew that the LHW ‘*came from the GP practice’*, patients consistently contrasted their LHW positively with other practitioners they had experienced, as Additional files 1, 2 and 3 illustrate. Specifically, LHWs transcended physical and mental health problems, whereas GPs were limited to physical health, and counsellors or psychological therapists to mental health. Paradoxically, patients described being able to engage more readily with psychological issues in this context than in previous relationships that had been explicitly psychotherapeutic (see Additional files 1 and 3). Patients felt personal engagement and a sense of responsibility for their lives in their relationship with LHWs that they did not recount with other practitioners.

**Table 1:** **Main types of help that patients described the LHWs providing**

Type of activity	Number of patients describing activity*	Examples
Signposting of other services	19	Directing patients to health trainers, complementary practitioners, disability taxi service, ‘good neighbour’ scheme, local support groups and charities; providing other contact telephone numbers.
Education and information	19	Explaining COPD; providing worksheets and leaflets about COPD; information on coping strategies to support activities of daily living.
Health behaviour advice	18	Behavioural advice on smoking cessation; exercise and diet plans; accompanying patient in exercise; advice on sleep; collaborative goal setting; teaching relaxation techniques and providing relaxation CD.
Listening	17	Listening to patients’ accounts; providing ‘company’; talking informally about patient’s life; having tea with patient.
Supporting applications	9	Helping complete applications for state benefits, bus passes and disability parking badges; supporting other applications including for grant for headstone for patient’s relative, and applications for volunteering and employment.
Providing a positive perspective	8	Encouraging patients; rewarding patients’ achievement with praise or small gifts; suggesting new ways of thinking about problems.
Practical support	7	Liaison with local services to effect practical adaptations in the home such as hand rails, fire alarms and chair supports; accompanying patients to local services or groups.
Formal cognitive-behavioural intervention	7	Management of anger, panic and sleep problems; addressing low mood; showing connection between mental and physical health.

*All patients described more than one activity.

### Listening to me

Although patients described the LHWs as authoritative and ‘*knowing her job’*, no-one described them as setting the intervention agenda. Patients characterised initial encounters with the LHW as being patient-centred. For example, G8 contrasted having expected ‘*someone … kind of preaching [health advice] to me’* to the experience of a ‘*very, very good listener … who was interested in me and my health’.* When describing the LHWs’ intervention, patients therefore recounted different types of help that reflected the diversity of their social, emotional and clinical needs (Table 1). Patients did not, however, describe the LHWs as non-directive; but the direction provided was based on having listened to the patient. One described how ‘*she can ask you a load of questions and then steer you, albeit quite often you don’t realise she's steering you, along a certain path*’_R70_. Others recounted the LHWs ‘*suggesting’* actions, or ‘*offering’* help. LHWs modified their suggestions based on patients’ experience; for example one LHW devised a home-based exercise programme after a patient rejected group exercise.

### Relating to me

Patients had generally anticipated that the LHW would be a ‘*nurse that could tell you more about COPD’*_R70_ or ‘*a nurse that was just checking up*’_B143_. By contrast, they experienced a practitioner responding to patients’ needs rather than professional priorities. Patients were surprised and appreciative that the LHWs supported claims for state benefits, arranged access to community resources such as support groups, or joined in their exercise activities. For example, one recalled that the LHW *‘came to see me when I was in the [respite home, after the LHW had discharged her]… She was very good coming to see me, and she didn't have to do, really*’_B143_. They consistently described the LHWs as ‘*like a friend’* and ‘*caring’* and recalled ‘*enjoying’* time together, even to extent of having ‘*fun’*. Most (17/28) initial consultations were in patients’ homes, which was critical to patients’ sense of personal relationship: ‘*It’s like sitting down with my sister or a neighbour … she’d come in, take her coat off, sit down, get a cup of tea… she was natural*’_B147_.

### Investing in me

Patients consistently described the LHWs as ‘*positive’.* This did not mean optimistic; rather, it signified valuing patients’ capacity for improvement. For example, patients described the LHW being ‘*pleased when I said I’d go to the gym*’_B137_ or being *‘so proud of you’*_B147_ when a patient succeeded in climbing stairs. That is, patients felt encouraged and motivated by the sense that the LHWs valued them and their attempts to change. G8 explained that ‘*I felt she [LHW] was investing in me … and I didn’t want to let her down’ ,* whereas with his GP ‘*I just feel I’m one of another list of people’.* Recounting that the LHW offered to accompany him exercising, he felt: *‘Here’s somebody’s taking an actual personal interest in my health and, you know … I would let myself down and her down if I hadn’t done everything I said I would do…I found it a bit more motivational … inspirational, and it wasn’t just a case of…“Here’s a little booklet about keeping fit, read that”.’*

### Deviant cases: not for me

A few patients were negative about what the LHWs could achieve, including those who declined or withdrew from the intervention, and one who completed it. By contrast with the positive patients, who generally described not knowing what to expect from the LHW, each of these described strong preconceptions about the LHW role and its futility. The patient who completed the intervention had professional experience of cognitive-behavioural therapy (CBT), which she saw as central to the LHWs’ role and which previous experience had left her sceptical about: ‘*The CBT didn’t do it for me, but I think I’d already made up my mind about that before I went to the surgery …I think it’s a bit of a waste of time’*_G22_. A patient who declined explained that *‘I don’t want anybody to help us. We’ve passed the stage, nobody can help, I am convinced of that*’_R34_.

### Practice staff experiences: compartmentalisation of holistic care

Practice staff members were uniformly positive about the LHWs. They described LHWs as *‘an opportunity to sort of look after people in a truly holistic approach’*_B2GP_ or ‘*improving whole-person care’*_G8GP_. A GP explained that *‘I wanted to get him [patient referred to LHW] to control his condition better, so to take the reins a little bit more’*_G8GP_. Similarly, a HCA recalled referring a patient who was depressed because ‘*there might be something she [patient] could do, could look at you know, things that you could change*’_R1HCA_.

However, staff described these areas of care as peripheral to normal clinical practice for both GPs (‘*if you just had a fifteen-minute consultation and you're asking at the very end if you're depressed …if they say “yes” you've just opened up a can of worms and you can't deal with properly at the time*’_R4GP_) and nurses (‘*We are very aware of their social needs and their psychological needs but, with the best will in the world, it is a time issue’*_B1PN_). Practitioners could say little about the LHWs’ activities and referred few patients to them (GPs referred 8; PNs and HCAs referred 20), commenting that they had *‘not really made a great difference to my workload or my role really’*_R1HCA_*.* The LHWs were assimilated into the fragmented nature of care: ‘*[The LHWs]’d come back with all their bits and use the computers, but of course we’re all in our own little bubbles, aren’t we, our own little rooms, getting on with stuff*’_G9PN_. Indeed, this seemed key to practices’ acceptance of the LHWs. As a practice manager explained: ‘*They just, like, fitted in, yeah. Yeah, they’re just there. Which I suppose is a compliment because they just, they do fit in and you know, they know their role that they’re doing … I don’t need to give loads of my time*’_G1ADMIN_.

## Discussion

### Summary of findings

Almost all patients saw the LHWs as providing a new type of care, which they were enthusiastic about. Patients described how the intervention differed from that experienced from other physical or mental health practitioners. They experienced the intervention as holistic in matching their experience of the interconnectedness of emotional and physical needs, and they contrasted it with compartmentalised care from other practitioners. The intervention was not explicitly psychological, in that LHWs did not primarily use psychotherapeutic techniques. Nevertheless, it was psychological in its effect, particularly in motivating patients to engage in self-management. Patients knew that this was a short term, time-limited intervention and their accounts showed no evidence of needing continued contact with LHWs. That is, the intervention was experienced as empowering and motivating rather than simply supportive. A few patients rejected the offer of the intervention or withdrew from it, but these had approached it with attitudes based on previous psychotherapeutic interventions which they saw it as replicating and which they were negative about.

Practice staff were pleased that the LHWs addressed patients’ psychosocial needs but regarded these needs, and therefore the LHW intervention, as peripheral to, and separate from, physical care, which was their main priority. Therefore, LHWs were accommodated by – rather than integrated into – practices.

### Comparison with previous literature

The key elements of the intervention were the sense of relationship that patients felt with the LHW, the collaborative rather than directive role that the LHWs took, and the personal investment that patients felt from the LHWs. These elements correspond strikingly to the three conditions that foster internal motivation according to Self-Determination Theory (SDT): ‘competence’ , ‘relatedness’ and ‘autonomy’ [19]. By listening to patients and being directed by patients’ needs rather than professional agendas, the LHWs respected patients’ *competence* to choose priorities and to make decisions about their health and psychosocial needs; by relating to patients holistically as individuals rather than by focusing on specific categories of physical or psychological need, the LHWs demonstrated *relatedness*; and by investing in the patients’ capacity to change, the LHWs fostered their *autonomy*. In Self-Determination Theory, intrinsic motivation is undermined by reliance on external goals or surveillance, even in the context of positive feedback [20]. Therefore, we suggest that the contrast that patients described between LHWs and other practitioners arose in part from the LHWs’ promotion of internal motivation for behavioural and psychological change, whereas other practitioners had undermined internal motivation by emphasising practitioners’ goals in the context of health surveillance. The importance of internal motivation in the present context is that it outlasts the specific circumstances that promote it; in the present study, patients’ accounts suggested that the motivational effect of the LHW persisted after the final contact with her.

That LHWs and their intervention did not become integrated into practices is consistent with the experience of previous studies in primary care [12, 16]. Similarly, the Improving Access to Psychological Therapies (IAPT) programme has overseen extensive provision of psychological practitioners for primary care patients in England, but these work separately from primary care practitioners [21]. Despite claims that primary care should be the site of holistic patient care, compartmentalisation of physical and psychological care seems to be engrained in UK primary care [12]. Take-up by practices of an intervention designed to address psychosocial aspects of long-term conditions therefore seems to depend on it being accommodated within this fragmentation of care, even to the extent of the irony that ‘holistic care’ becomes the domain of one, relatively peripheral, section of the primary care team.

### Strengths and limitations

While novel, the intervention we developed was based on evidence about the need for an integrated approach to psychosocial and physical needs, and the patients recognised that this integration was novel in their experience of care. The key strengths of our evaluative method are that we characterised the intervention through the experience of those receiving it, and that we obtained staff as well as patient perspectives.

The implementation of our intervention and evaluation both have limitations. We trained only two LHWs, who were highly experienced practitioners. Although we saw no differences between patients’ accounts of each, it remains unclear how patients would experience the intervention if implemented by a larger pool of practitioners. Similarly, the LHWs worked in only 3 practices - too few to be representative of general practice. Our evaluation was limited by minimal recruitment of patients who declined the intervention or withdrew from it to the qualitative study. Therefore our detection of the reasons for non-participation was very limited. The follow-up period was short and we do not know whether the benefits that patients reported would be sustained in the absence of further LHW involvement.

## Conclusion

The LHW intervention has potential, and should be evaluated formally for its ability to enhance motivation for self-care and to address psychosocial needs in patients with COPD and other LTCs. It will be important to measure patient-level outcomes and long-term effects and to identify what kind of continuing support may be necessary for long-term benefit. If the intervention is effective, there would be broader implications also. First, brief and discontinuous relationships have potential in primary care. The motivational success of the LHW-patient relationship suggests that the type of the relationship and the work done in the relationship may be more important for fostering self-management than continuity with a named clinician over time. Secondly, a whole-systems approach may not be necessary to facilitate patient-centred care in primary care. Expecting a stronger degree of integration of an intervention such as this may be unrealistic given the culture of UK primary care [22].

Nevertheless, it will be valuable to consider whether there are potential benefits for the existing primary care workforce in adopting elements of the LHW intervention, particularly using SDT in their interactions with patients, and integrating care of both physical and psychosocial needs in order to enhance patients’ motivation for self-management.

## Electronic supplementary material

Additional file 1: **R65 – Patient needs vs professional remits.** (DOCX 16 KB)

Additional file 2: **G81 – Integrated vs compartmentalised care.** (DOCX 16 KB)

Additional file 3: **B147 – Psychological engagement in the context of physical care.** (DOCX 17 KB)

## Abbreviations

(CBT): Cognitive-Behavioural Therapy
(CHOICE): Choosing Health Options In Chronic care Emergencies
(COPD): Chronic Obstructive Pulmonary Disease
(GP): General Practitioner
(HCA): Healthcare Assistant
(HCP): Healthcare Professional
(IAPT): Improving Access to Psychological Therapies
(LHW): Liaison Health Worker
(LTCs): Long-term conditions
(PIS): Patient information sheet
(PN): Practice Nurse
(QOF): Quality and outcomes framework
(QP): Quality and productivity
(SDT): Self-Determination Theory.
