# Supplementary material for: A motivational intervention for patients with COPD in primary care: qualitative evaluation of a new practitioner role
Source: BMC Fam Pract. 2014 Oct 6;15:164. doi: 10.1186/1471-2296-15-164 (PMC4286939; doi:10.1186/1471-2296-15-164)
Supplement: Supplementary file 1 — Additional file 1: R65 – Patient needs vs professional remits. (DOCX 16 KB) [file 12875_2014_1138_MOESM1_ESM.docx]

Additional file 1

R65 – Patient needs vs professional remits

Background

50 year old woman with COPD, osteoporosis, and depression and recovering from alcohol abuse. Saw LHW four times. The LHW: used cognitive methods to address sleep and mood; provided distraction techniques to control worry (colouring drawings); and helped with application for welfare benefits.

Comparison with other practitioners

R65 contrasted the LHW’s willingness to address the breadth of her problems with other practitioners’ narrow remits: ‘She listened, and she didn’t say “No, no”… If you say something to some professionals “Oh, right, that’s not my side of it and it’s nothing to do with me”, because really my benefits weren’t affecting what she’d come for… She sort of like took over the benefits sort of it, which really is not her job, is it, really?’. When asked if she had sought the GP’s help for depression, R65 said: ‘No. I told him about, but I don’t think he can much He said … “Go and see this person and go and see that person.”’

R65 felt that the GP had nothing more to offer: ‘He said I’m on the best medication, basically he can’t help me anymore.’ By contrast, the LHW was’really, really helpful … by the time she’d finished, I’d improved a great deal and that was just through talking it through with [LHW] and her sort of understanding me’.
