# Supplementary material for: A motivational intervention for patients with COPD in primary care: qualitative evaluation of a new practitioner role
Source: BMC Fam Pract. 2014 Oct 6;15:164. doi: 10.1186/1471-2296-15-164 (PMC4286939; doi:10.1186/1471-2296-15-164)
Supplement: Supplementary file 2 — Additional file 2: G81 – Integrated vs compartmentalised care. (DOCX 16 KB) [file 12875_2014_1138_MOESM2_ESM.docx]

Additional file 2

G81 – Integrated vs compartmentalised care

Background

58 year old woman with COPD, bipolar disorder and history of alcohol abuse, currently reporting panic, fibromyalgia and ‘ME’. She saw physical and emotional problems as intimately linked: ‘Where my physical health is not good it has an effect on my mental health. When my mental health is not good it has an effect on my physical health’. Saw LHW four times. The LHW: provided cognitive strategies to manage panic; referred her to Expert Patient Programme; encouraged exercise.

Comparison with other practitioners

While positive about the GP, who ‘does everything she can’, her role had ‘generally been medication … I think they [GPs] are much more informed about physical health than they are mental health.’ For mental health and self-management the GP offered only information: ‘She did mention this expert patients scheme … and she did download some information about ME’. By contrast, the LHW emphasised the patient’s own responsibility: ‘It's [LHW intervention] about reminding me that I'm responsible for my health, and that the GP is there to help when you need that, but there's a lot you can do for yourself’. The patient also saw a PN but described this as ‘being monitored and medication’.

The patient did not see the LHW as a mental health intervention. Compared with previous counselling, the LHW ‘was about COPD, so it's about physical health …seeing a counsellor was more about my mental health’. She contrasted engaging with the LHW to the boundaried nature of counselling: ‘It's not like [LHW] would come at a certain time and stay for an hour. It could be less than an hour, it could be more than an hour … Those boundaries weren't there … How it differs from counselling is I was being offered stuff, which a counsellor can't do. She can't, for instance, wouldn't go swimming with you or they wouldn't find out about this expert patient thing’.
