# Supplementary material for: A motivational intervention for patients with COPD in primary care: qualitative evaluation of a new practitioner role
Source: BMC Fam Pract. 2014 Oct 6;15:164. doi: 10.1186/1471-2296-15-164 (PMC4286939; doi:10.1186/1471-2296-15-164)
Supplement: Supplementary file 3 — Additional file 3: B147 – Psychological engagement in the context of physical care. (DOCX 17 KB) [file 12875_2014_1138_MOESM3_ESM.docx]

Additonal file 3

B147 – Psychological engagement in the context of physical care

Background

61 year old woman with panic anxiety. Saw LHW four times. The LHW: addressed anxiety (relaxation and distraction exercises); advised on inhaler technique; liaised with other services for alterations to B147’s house; helped application for welfare benefits; referred to complementary therapy.

Comparison with other practitioners

In explaining why she did not discuss her anxiety with the GP, B147 explained that ‘All he does is give me pills … He doesn’t really talk, don’t you know. He talks but you don’t seem to get anywhere … I’ll try you on these pills, I’ll try you on these pills’. By comparison, with the LHW’it’s like sitting down with my sister or a neighbour, you know what I mean? Because [LHW]’d come in, take her coat off, sit down, get a cup of tea, it was just, she was natural… there was no embarrassment … no awkwardness’.

B147 also contrasted the LHW with a counsellor or psychotherapist she had seen previously at the GP practice: ‘It was sort of the same thing [as the LHW] but it was I had to go in a room and it drove me nuts… She [psychotherapist] never explained it very well and I really hated going … it’s the same kind of line of work as what [LHW] done, but I understood hers but I didn’t understand [therapist]’. By contrast, the LHW’s intervention ‘made sense’, so that ‘I know where my aim is … now… She opened me up, opened my eyes a wee bit, you could say, inspiration.’

Patients typically described being changed by LHW involvement. Their language, such as ‘[She] broke this stalemate’_R195_, and ‘[LHW] made me understand’_G89_ consistently portrayed the LHW as achieving psychological change and as prompting motivational and behavioural changes that patients then took responsibility for. For example,‘[LHW] eased you along the route for you to accept that you have got problems … made me think about what I needed to do’ _R70_, or ‘I was in this stalemate and I wasn’t kind of accepting or probably refusing to accept things that was wrong … She kind of clawed away a lot of the rubbish that was going around in my mind … She kind of gave me this push that set a lot of things rolling’_R198_. Further analysis suggested three elements of LHWs’ intervention that underlay their ability to instigate psychological and behavioural changes in their patients.
